# Parthanatos drives cognitive decline in repeated brain trauma: MSC-derived exosomes as a novel therapeutic strategy

**DOI:** 10.3389/fphar.2025.1622018

**Published:** 2025-09-02

**Authors:** Heba Mohammed Refat M. Selim, Amira A. El-Gazar, Dalaal M. Abdallah, Hagar B. Abo-Zalam, Ghada M. Ragab, Ahmed N. Abdallah, Rabab A. El-Gazar, Sultan Alshehri, Einas M. Yousef, Rayan Ballal, Sahar N. Aljarallah, Asmaa Saleh, Nada F. Abou Chahin, Naheda S. Alsammak, Rasha A. Mandil, Hanan S. El-Abhar

**Affiliations:** ^1^ Department of Pharmaceutical Sciences, College of Pharmacy, AlMaarefa University, Riyadh, Saudi Arabia; ^2^ Department of Pharmacology and Toxicology, Faculty of Pharmacy, October 6 University, Giza, Egypt; ^3^ Department of Pharmacology & Toxicology, Cairo University, Cairo, Egypt; ^4^ Department of Pharmacology and Toxicology, Faculty of Pharmacy, Misr University for Science and Technology, Giza, Egypt; ^5^ Hormones Department, Medical Research and Clinical Studies Institute, National Research Centre, Cairo, Egypt; ^6^ Department of Clinical Pharmacy, October 6 University, Giza, Egypt; ^7^ Department of Pharmaceutics, College of Pharmacy, King Saud University, Riyadh, Saudi Arabia; ^8^ College of Medicine, Alfaisal University, Riyadh, Saudi Arabia; ^9^ Department of Pharmacy Practice, College of Pharmacy, AlMaarefa University, Riyadh, Saudi Arabia; ^10^ Department of Pharmaceutical Sciences, College of Pharmacy, Princess Nourah bint Abdulrahman University, Riyadh, Saudi Arabia

**Keywords:** exosomes, parthanatos, cyclophilin B, PARP1, calpain, oxidative stress, NAD +

## Abstract

**Introduction:**

Repetitive traumatic brain injury (RTBI) represents a cumulative neurological insult associated with progressive neurodegeneration and limited therapeutic options. In this study, we uniquely evaluate the neuroprotective potential of mesenchymal stem cell (MSC)-derived exosomes in a rat model of RTBI, an area scarcely explored.

**Methods:**

RTBI was induced via a controlled mechanical impact to the skull once every day for 5 days. MSC-derived exosomes were administered 24 h after the final insult in two paradigms: a single dose (MSC-Ex1) with 2 weeks of follow-up, and a dual dose (MSC-Ex2) given 1 week apart, with sacrifice 1 week later. Rats were assigned to four groups: control, RTBI, RTBI + MSC-Ex1, and RTBI + MSC-Ex2.

**Results:**

MSC-derived exosome regimens comparably restored cognitive performance in the Novel Object Recognition and Y-maze tests. While both treatment paradigms preserved cortical histoarchitecture, the double-dose regimen led to a more pronounced restoration compared to the moderate tissue recovery observed in the single-dose group. Crucially, this work identifies parthanatos inhibition as a novel mechanistic axis for MSC-derived exosomes-mediated neuroprotection. MSC-derived exosomes attenuated excitotoxicity and oxidative stress, quelling the parthanatos cascade by suppressing PARP1, PAR polymers, nuclear AIF and MIF, as well as calpain, key executors of this caspase-independent cell death pathway. Additionally, MSC-derived exosomes normalized cyclophilin B and Hsp70 levels, suggesting their compensatory role in modulating the endogenous stress response.

**Conclusion:**

Overall, these findings demonstrate that MSC-derived exosomes counteract RTBI-induced neurodegeneration through multifaceted mechanisms, with parthanatos suppression at the core. Importantly, the dual-dosing regimen conferred no significant benefit over the single dose, highlighting the therapeutic promise of early intervention. This study positions MSC-derived exosomes as a novel, cell-free therapy capable of intercepting RTBI-induced neuropathology by targeting an under recognized form of programmed cell death.

## Graphical Abstract

**Figure:**
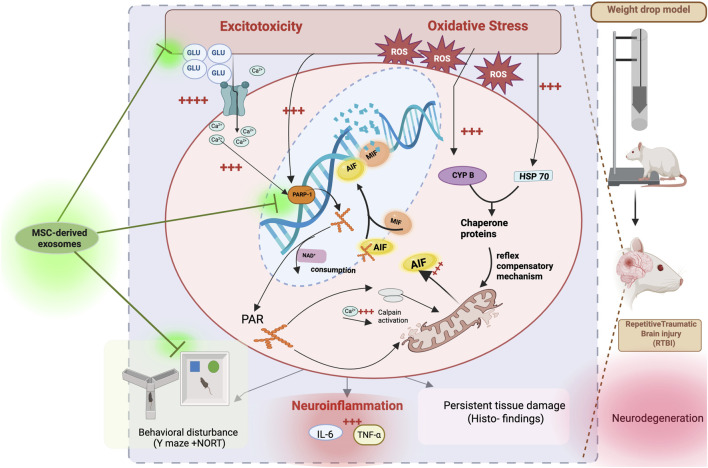
Diagram depicting neurodegeneration pathways after repetitive traumatic brain injury (RTBI) from weight drop model in rats. Pathways include excitotoxicity, oxidative stress, neuroinflammation, and parthanatos cell death. MSC-derived exosomes potentially modulate these effects, impacting behavioral disturbances and histoarchitecture. Components like GLU, ROS, PARP-1, and proteins like AIF, MIF, and calpain, Cyp P, and HSP70 are shown interacting within these mechanisms, ultimately leading to tissue damage and compensatory responses.

## 1 Introduction

Advancements in traumatic brain injury (TBI) research are driving the emergence of novel therapeutic strategies, especially in light of the lack of curative or disease-modifying treatments for the long-lasting neurological deficits associated with TBI (Naffaa and Naffaa, 2025). As a neurodegenerative condition, TBI is pervaded by widespread neuronal loss mediated through multiple cell death pathways, culminating in discernible pathological behavioral and motor phenotypes (Brett et al., 2021). Among the chief secondary injury mechanisms in TBI are excitotoxicity and oxidative stress (OS), which significantly contribute to DNA damage (Baracaldo-Santamaría et al., 2022; Smith et al., 2013) a crucial factor in promoting neuronal demise by activating various cell death scenarios (Makarevich et al., 2020).

Notably, substantial DNA damage can occur within minutes of the initial injury and persist for days. While intrinsic DNA repair mechanisms are promptly triggered, DNA lesions are frequently observed in TBI (Baracaldo-Santamaría et al., 2022; Smith et al., 2013). Previous studies have shown that DNA fragmentation can persist when the repair machinery is overwhelmed or rendered ineffective by repetitive insults, leading to sustaining functional impairments and progressive neurodegeneration (Davis and Vemuganti, 2021; Welch and Tsai, 2022). This fact underscores that endurance of DNA damage appears to be more pronounced in models of repetitive TBI (RTBI) than in single-impact injuries. Consequently, therapies aimed at minimizing DNA damage and enhancing repair capacity may improve post-TBI recovery outcomes (Davis and Vemuganti, 2021).

Poly (ADP-ribose) polymerase-1 (PARP1) serves as a genomic sentinel, detecting and initiating repair of DNA lesions (Ray Chaudhuri and Nussenzweig, 2017). Nevertheless, hyperactivation of PARP1 unleashes a pathological cascade whereby its product, poly-ADP ribose (PAR), escapes the nucleus, instigating a caspase-independent programmed cell death pathway known as parthanatos, an indispensable player of the pathophysiology of numerous diseases (Fatokun et al., 2014). Extracellular PAR inhibits mitochondrial hexokinase, causing bioenergetic collapse and depleting nicotinamide adenine dinucleotide (NAD^+^) (Andrabi et al., 2014; Fouquerel et al., 2014). In parallel, PAR also interacts with mitochondrial oxidoreductase apoptosis-inducing factor (AIF), prompting its release and translocation to the nucleus (Dawson and Dawson, 2017; Y. Wang et al., 2011). Prior nuclear entry, AIF complexes with macrophage migration inhibitory factor (MIF), and together, they traverse to the nucleus, culminating in the terminal phase of the parthanatos cascade, characterized by genomic DNA fragmentation into segments.

In addition to apoptosis, necroptosis, pyroptosis, and ferroptosis (Dixon et al., 2012; El-Gazar et al., 2024; Raghupathi, 2004), parthanatos represents a unique, NAD^+^ depleting, DNA-damage–driven mode of cell death. This underexplored machinery may be particularly relevant in RTBI, where cumulative oxidative and genotoxic insults persist beyond the acute phase. Although hindering parthanatos trajectory has been proposed as a promising therapeutic avenue for a range of disorders (Wang et al., 2016), including TBI (Sun et al., 2022) its relevance in RTBI has not been addressed, despite the heightened susceptibility of this repeated insult to activate sustained cell death pathways. Potential of this repeated insult to activate sustained cell death signaling pathways.

RTBI, often observed in contact sports and military populations, is characterized by unresolved secondary injury cascades, including persistent oxidative stress and DNA damage (Davis and Vemuganti, 2021; Janković and Pilipović, 2023), which may drive parthanatos activation more robustly than in single-impact models. While prior studies have implicated PARP-1 in TBI (Loane et al., 2015; Zhang et al., 2025), the full parthanatos cascade has yet to be comprehensively explored in the context of RTBI. This is particularly critical, given that such injuries, despite lacking overt initial symptoms, are increasingly recognized as a public health concern due to their contribution to progressive cognitive and behavioral decline and their strong association with neurodegenerative conditions like chronic traumatic encephalopathy (CTE) (Brett et al., 2021; Mckee and Daneshvar, 2015).

Unlike traditional cell-based therapies, extracellular vesicles (EVs) have emerged as promising therapeutic agents for brain disorders due to their ability to cross the blood–brain barrier (BBB), deliver bioactive molecules and enhance intercellular communication (Kumar et al., 2024). Among the various types of EVs, exosomes represent a cell-free therapeutic approach that circumvents the challenges of cell survival, engraftment, and immunogenicity, while retaining robust paracrine regenerative and neuroprotective potential (Huber and Wang, 2023; You and Ikezu, 2019). The modulatory and protective capacities of exosomes largely depend on their biological origin and molecular composition (Kalluri and LeBleu, 2020; J. Wang et al., 2022). In this regard, mesenchymal stem cell (MSC)-derived exosomes have demonstrated wide-ranging therapeutic potential across several disease models by promoting intracellular communication, enhancing tissue regeneration, and mitigating disease progression (Kalluri and LeBleu, 2020; Nikfarjam et al., 2020; Zargar et al., 2022).

Although several recent studies have highlighted the neuroprotective effects of exosomes in TBI, their therapeutic efficacy in the context of RTBI, a condition marked by accumulated redox imbalance and unresolved DNA breakage, remains largely unexplored. Unlike single-impact TBI, RTBI may preferentially engage the caspase-independent, DNA damage–driven cell death pathway parthanatos, amplifying neurodegeneration. In this study, we aim to investigate the neurotherapeutic potential of MSC-derived exosomes in a model of RTBI, with a particular focus on their ability to disrupt parthanatos activation and modulate its downstream molecular cascade. By targeting this underexplored mechanism, the study seeks to establish MSC-derived exosomes as a promising, cell-free intervention for mitigating RTBI-induced neuronal injury.

## 2 Materials and methods

### 2.1 Exosomes isolation and quantification

#### 2.1.1 Isolation of MSC-derived exosomes

Conditioned medium was harvested from the supernatants of previously cultivated third passage rats bone marrow-derived MSC cultures. Subsequently, upon achieving confluence, the medium was substituted with serum-free DMEM and cultured for a further 48 h to harvest the conditioned medium containing exosomes. The conditioned medium was subsequently centrifuged first at 300 × g for 10 min to remove cell debris, then the supernatant was centrifuged at 2,000 × g for 20 min, followed by 10,000 × g for 30 min to pellet larger vesicles. The supernatant was collected and ultracentrifuged at 100,000 × g for 70 min to isolate exosomes. After discarding the supernatant, the exosome pellet was resuspended in PBS, washed in serum-free Medium 199 containing 25 mM HEPES (Sigma-Adrich, MA, United States), and subjected to a second ultracentrifugation under the same conditions. The purified exosomes were incubated overnight in the collection medium before storing the final pellet at −80 °C (Abdallah et al., 2024).

#### 2.1.2 Characterization of MSC-derived exosomes

The total protein content of the exosomes was measured by BCA protein assay kit (Sigma-Aldrich. The isolated exosomes were diluted in PBS in ratios of 1:10 (by 10-fold) and mixed with BCA reagent and incubated at 60 °C for 15 min, followed by recording the related absorbance at 562 nm using NanoDrop™ spectrophotometer (ND-1000, Thermo Fisher Scientific, CA, United States). The standard curve was drawn by performing the same procedure for different concentrations (50–250 μg/mL) of bovine serum albumin (Szatanek et al., 2011).

#### 2.1.3 Transmission electron microscopy (TEM)

A sample was prepared by placing a drop of the exosome suspension onto a copper grid and allowing it to air dry. It was then stained with uranyl acetate and examined under a TEM to assess morphology and size. Images were acquired using secondary electron at a working distance of 15–25 mm and an accelerating voltage of 20–30 kV. Digital acquisition and analysis were conducted using the JEOL IT300 system (Tokyo, Japan).

#### 2.1.4 Multiparametric characterization of exosomes by flowcytometry

MSC-derived exosomes isolated from cell culture supernatants were characterized using multiparametric flowcytometric analysis with a panel of specific markers: CD9 and CD63 (exosomal membrane markers), along with CD45 (a stem cell marker) (Theodoraki et al., 2021). Exosomes at a concentration of 1 × 10^10^/mL were suspended in 100 µL of filtered PBS containing 2% exosome-depleted fetal bovine serum (FBS), supplemented with protease and phosphatase inhibitors, and subsequently stained using a panel of three primary antibodies. The monoclonal antibodies used for exosome staining, anti-CD9 (MM2/57), anti-CD63 (MEM-259), and anti-CD45 (30-F11), were procured from Thermo Fisher Scientific and all conjugated to fluorescein isothiocyanate (FITC). For exosome staining, 15 µL of the exosome suspension was mixed with 5 µL of a 1:100 diluted antibody solution and incubated for 45 min at room temperature in the dark. Following, exosomes were washed using a washing buffer composed of 0.2 µm-filtered PBS supplemented with 2% exosome-depleted FBS (Thermo Fisher Scientific). Finally, the exosomes were analyzed by flowcytometry. Following sample processing, data was acquired using flowcytometry and cell populations were gated based on monoclonal antibody labeling. Laser power settings were either optimized to ensure fluorophore intensities remained within the detection range or operated at maximum power (405 nm: 175 mW; 488 nm: 145 mW; 561 nm: 90 mW; 642 nm: 145 mW). FITC fluorescence, corresponding to CD45, CD9, and CD63 staining, was detected in channel 2 using a 480–560 nm filter. All measurements were taken at ×60 magnification under low flow conditions. Data was analyzed using Navios EX Software (BE14548). Positive exosome counts were determined as the fraction of EVs positive for each marker relative to the total EVs captured using anti-CD9, CD63, and CD45 staining. Parametric plots from the flowcytometry data displayed the percentage of each cell population within the gated events. Exosomes were defined as CD9^+^, CD63^+^, and CD45^-^.

#### 2.1.5 Dynamic light scattering (DLS)

The exosome suspension was diluted in PBS to measure the size distribution using a DLS instrument (Zetasizer Nano ZN, Malvern Panalytical Ltd., WA, United Kingdom) at a fixed angle of 173° at 25 °C. Triplicate measurements were conducted for each sample. The same equipment was used for the determination of zeta potential, and the data was analyzed to determine the average size and polydispersity index (PDI) of the exosomes.

### 2.2 Animal handling

The permit number PT 3866 was granted by the Research Ethics Committee of the Faculty of Pharmacy, Cairo University (Cairo, Egypt), approving the current protocol in accordance with the 2011 NIH Guide for the Care and Use of Laboratory Animals. Adult male Sprague-Dawley rats weighing between 250 and 300 g were supplied from the National Research Centre (NRC, Giza, Egypt) and were left 1 week for acclimatization to the new habitat (24 °C ± 2 °C, 12-h light/dark cycle, controlled ventilation) with unrestricted access to food and water.

### 2.3 Study design and repetitive trauma induction

Animals (n = 9/group) were randomly stratified into four cohorts. The first, designated as the negative control group, was subjected solely to inhalational anesthesia and intraperitoneal administration of normal saline (used as the vehicle for exosomes) for seven consecutive days. The remaining animals underwent RTBI as delineated in our prior investigations (El-Gazar et al., 2019; Soubh et al., 2021). Succinctly, anesthesia was induced using isoflurane vaporized at 4% concentration with a flow rate of 1–2 L/min. Once the rats reached a surgical plane of anesthesia, confirmed by the loss of righting reflex and absence of response to toe pinch, they were transferred to the weight-drop model platform. While anesthesia was maintained with 1.5% isoflurane at the same flow rate, a 75 g sharp-edged weight was released from a height of 25 cm onto the right anterior frontal region of the skull once daily for five consecutive days (Albert-Weißenberger et al., 2012; El-Gazar et al., 2019; Flecknell, 2009). Based on behavioral observations, absence of skull fracture or extended unconsciousness, and consistency with prior literature (El-Gazar et al., 2019; Soubh et al., 2021), the severity of the induced injury is characterized as mild injury.

Following RTBI induction, animals were distributed as follows: (1) RTBI group, left untreated for 2 weeks post-injury; (2) RTBI + MSC-Ex1, administered a single intravenous (I.V.) dose of exosomes (100 µg/rat) 24 h post-final trauma and observed for 2 weeks; (3) RTBI + MSC-Ex2, received two I.V. doses of exosomes (100 µg/rat), first at 24 h and again at 7 days post-trauma, then monitored for one additional week. Exosomes were prepared at a concentration of 200 μg/mL, and each rat received 0.5 mL (equivalent to 100 µg per rat). The initial treatment paradigm (24 h post-injury) was adopted based on prior studies advocating early exosome administration to mitigate secondary brain damage (Ni et al., 2019; Williams et al., 2020; Xiong et al., 2017) whereas the extended regimen (24 h and 7 days post-injury) was informed by a prior investigation on distal middle cerebral artery occlusion in aged rats (Dumbrava et al., 2021).

### 2.4 Behavioral examinations

#### 2.4.1 Novel object recognition test (NORT)

This test is used to assess visual recognition memory facilitating the comparison of previously stored information with current stimuli (Grayson et al., 2015). Interruption in this test may indicate heightened anxiety, cognitive dysfunction, and/or neurological impairment, all of which could suppress the animal’s authentic exploratory behavior. The NORT was developed using rats, which inherently favor investigating novel objects over familiar ones (Becegato and Silva, 2022). In quiet environment, animals were accustomed to a wooden box measuring 40 cm × 40 cm with opaque arena’s walls. They underwent three distinct sessions over 3 days; habituation session (Day16): rats were permitted to explore the testing area devoid of any objects before the test; Training session (Day 17): the rats were allowed to explore two identical rectangular-shaped objects and to ensure that rats are no longer novelty-driven, and the environment had become familiar, they were allowed to explore the two identical rectangular objects for about 15 min in 3 sessions until the time spent on the two objects was almost identical. Testing session (Day 18): one of the training objects was replaced with a novel circular object of similar size (Brivio et al., 2020). The animal’s behavioral analysis was carried out using the ANY-maze video tracking system (Version 7.36, Wood Dale, United States), which quantifies the penchant and prejudice indices by tracking the exploration time of both familiar and the novel objects, as well as the freezing duration, potentially indicating the disrupted investigational inclinations. Thereafter, the discrimination index, preference score, absolute discrimination, novel object exploration %, and total freezing time were calculated.

#### 2.4.2 The Y-maze test

On the final day of the trial, the Y maze test was applied as a behavioral assessment of rats’ spatial reference memory, focusing on working memory, reference memory, and discriminative learning. The test measures rats’ willingness to explore new environments in woody Y shaped maze with three opaque arms; A, B, and C, arranged in a “Y” shape, typically connected at a central point. Animals were introduced to the center of the maze and allowed to freely explore the three arms (8 min). The maze was cleaned and deodorized using a 70% alcohol solution after each session (Danduga et al., 2018; Morgan et al., 2021; Mowaad et al., 2023). The recorded videos were analyzed using the ANY-maze video tracking system (Version 7.36, Wood Dale, United States), tracking number of alterations and total number of zone entries to calculate spontaneous alternation percentage (SA%).

### 2.5 Serum and tissue preparation

After behavioral assessment, animals were anesthetized with a substantial dose of thiopental sodium (100 mg/kg), and blood samples were collected from the femoral vein for sera preparation (El-Gazar et al., 2024; Flecknell, 2009). Subsequent to euthanasia, the entire brain was extracted from three rats per group and submerged in 10% neutral buffered formalin for microscopic evaluation. The right ipsilesional cerebral cortex of the remaining six animals was longitudinally divided into two sections. One specimen from each cohort of the six animals was homogenized in phosphate buffer for ELISA analysis, whereas the second portion was immersed in RNA later solution for qRT-PCR evaluation.

### 2.6 ELISA measurements

ELISA kits from AFG scientific (IL, United States) were used to determine the cortical contents of glutamate, and reactive oxygen species (Glut, #EK721837, 0.5–25 nmol/mL; ROS, #EK720415, 28–1600 pg/mL). Moreover, the cyclophilin B (CYP B), heat shock protein 70 (Hsp70), and MIF protein contents (CYP B, #ER0890, 3.125–200 ng/mL; Hsp70, #ER0890, 0.313–20 ng/mL; MIF, #ER0349, 0.156–10 ng/mL) were measured according to the provided kits from Fin-test (WUH, PRC). For the assessment of interleukin 1 beta (-1β, #ELR-IL1beta-001C, 3–300 pg/mL from Ray Biotech, GA, United States), calpain (#NBP2-74980, 0.63–40 ng/mL from Bio-Techne, MN, United States), AIF (#ELK5658, 0.16–10 ng/mL from ELK biotechnology, CO, United States), whereas and PAR (#MBS169573 from MyBioSource, CA, United States, 0.2–15 ng/mL) were measured and processed according to the provided instructions of each supplier. The NAD + cortical contents were determined using a colorimetric commercial kit supplied by Abcam (SH, PRC; #ab65348, 0.01–10 nmol/mg protein). Both AIF and MIF were determined after using nuclear extraction kits (Thermo Scientific, IL, United States; 78833) to determine their nuclear contents. The protein content of each sample was quantified using the Bradford technique (Bradford, 1976).

### 2.7 qRT-PCR technique

The cortical mRNA expression of PARP1, normalized to GAPDH, was determined using the 2^−ΔΔCT^ formula. The SV Total RNA Isolation System (Promega, WI, United States; #Z3101) was employed to extract total RNA, followed by processing the samples with an Invitrogen kit (CA, United States) for reverse transcription into cDNA. The qPCR method was conducted utilizing SYBR Green PCR Master Mix (Applied Biosystems, CA, United States; #4309155). The sequence of the primers and accession number for the target gene and the housekeeping gene GAPDH, procured from ThermoFisher Scientific (MA, United States), are listed in Table 1. To activate AmpliTaq DNA polymerase (ThermoFisher Scientific), the thermal protocol began with an initial incubation at 95 °C for 10 min. This was followed by 40 amplification cycles consisting of denaturation at 95 °C for 15 s and a combined annealing/extension step at 60 °C for 1 min. Fluorescence data were collected and analyzed using ABI Prism software, with quantification performed using the comparative threshold cycle (ΔΔCt) method via Sequence Detection Software version 1.7 (PE Biosystems, CA, United States). To ensure amplification specificity, melt curve analysis was performed after each qRT-PCR run, confirming the presence of a single, distinct amplification product. The amplification efficiencies of PARP1 and GAPDH primers were evaluated using standard curves generated from serial dilutions of cDNA. The calculated efficiencies (PARP1: 98.6%, GAPDH: 96.4%) were within the acceptable range, supporting the accuracy and validity of the ΔΔCt method for relative gene expression analysis.

**TABLE 1:** Studied genes, sequence, and accession number.

Gene symbol	Primers’ sequences from 5′ to 3′
*PARP1*	F: GAGTGGGCACAGTTATCGGC R: CCAGGCATTTCCAGTCTTCTCT NM_017076.2
*GAPDH*	F: CACCCTGTTGCTGTAGCCATATTC R: GACATCAAGAAGGTGGTGAAGCAG NM_001394060.2

GAPDH: glyceraldehyde-3-phosphate dehydrogenase; PARP1: poly (ADP-ribose) polymerase 1

### 2.8 Histopathological analysis and immunohistochemistry

Fixed brain specimens were sectioned, rinsed with water, dehydrated through increasing concentrations of ethyl alcohol, cleaned with xylene, and imbedded in paraffin for slicing into 4 μm thin sections utilizing a rotary microtome. The sections were affixed to slides and subsequently stained with hematoxylin and eosin (H&E) (Bancroft and Gamble, 2007). For IHC, brain sections were cut on adhesive slides, deparaffinized and re-hydrated, afterward a heat-induced epitope retrieval step was conducted, and tissue sections were incubated with primary anti-TNF-α (1:200, Santa Cruz, biotechnology, TX, United States) for an hour at room temperature. After washing, HRP-labelled detection kit (Bio SB, CA, United States) was used as manufacturer instructions to develop the color. Myer’s hematoxylin was used as counter stain. Positive expression was quantified as mean area percentage corresponding to each group.

### 2.9 Immunofluorescence technique

For preparation and deparaffinization, the formalin-fixed, paraffin-embedded (FFPE) tissue sections were fixed on slides at 60 °C for 40 min, deparaffinized using xylene (3×, 5 min), and rehydrated through graded ethanol followed by distilled water rinse. Following, slides were immersed in 10 mM sodium citrate buffer (pH 6.0) and heated in a pressure cooker (95 °C–98 °C, 10–20 min), then cooled at room temperature and rinsed in PBS. Non-specific binding was minimized by incubating sections with 5% BSA in PBS for 40 min in a humidified chamber. For primary antibody incubation, the sections were incubated overnight at 4 °C with anti-PARP antibody (Abfinity, Rabbit Oligoclonal, 1:200, Invitrogen, #RK242984) diluted in 1% BSA in PBS. Afterward, slides were washed 3× in PBS for 5 min to remove unbound primary antibody to be incubated with Alexa Fluor 488-conjugated secondary antibody (Goat anti-Rabbit IgG, 1:250, Invitrogen, #A32723) for 1 h at room temperature in the dark. Sections were washed 3× in PBS (5 min each in the dark), mounted with anti-fade medium, and stored at 4 °C protected from light. Finally, fluorescent imaging was performed using a LABOMED TCM400 microscope and Atlas 16 MP CMOS camera. Staining intensity was scored (0–3+) and H-score was calculated (0–300) based on Duplancic & Kero, (2021).

### 2.10 Statistical analysis

The findings are delineated as the mean ± standard deviation (SD), and statistical evaluations were executed using Tukey’s *post hoc* assessment following a one-way analysis of variance (ANOVA), considering significance at P < 0.05. Welch’s ANOVA, followed by Dunnett’s T3 multiple comparison *post hoc* test, was employed when variance heterogeneity was detected using the Brown-Forsythe test. Data visualization and statistical computations were carried out using GraphPad Prism (Version 9.0, CA, United States).

## 3 Results

### 3.1 Protein quantification of MSC-derived exosomes and their morphological characterization

**FIGURE 1:**
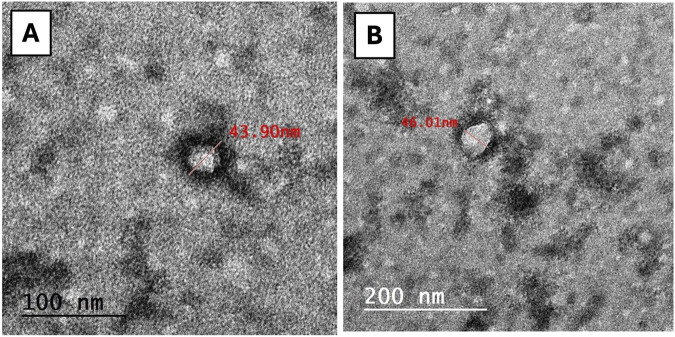
TEM image of exosomes. The exosomes expressing regular rounded architecture within the size not exceeding 200 nm (scale bar: **(A)** = 100 nm, **(B)** = 200 nm).

The results of the exosome characterization study revealed that the protein concentration of MSC-derived exosomes yielded a concentration of 200 ± 20 μg/mL. Moreover, the DLS analysis of the exosomes showed a uniform distribution of the particles, with an average diameter of 154 ± 72 nm and a PDI of 0.33, signifying the absence of aggregation. Additionally, as depicted in Figure 1 **,** the TEM images of the isolated exosomes revealed nanovesicles with the characteristic morphology and expected structure of exosomes. Their diameter ranged from 28.38 to 58.77 nm on 100 and 200 nm scales.

### 3.2 Identification and characterization of MSC-derived exosomes

**FIGURE 2:**
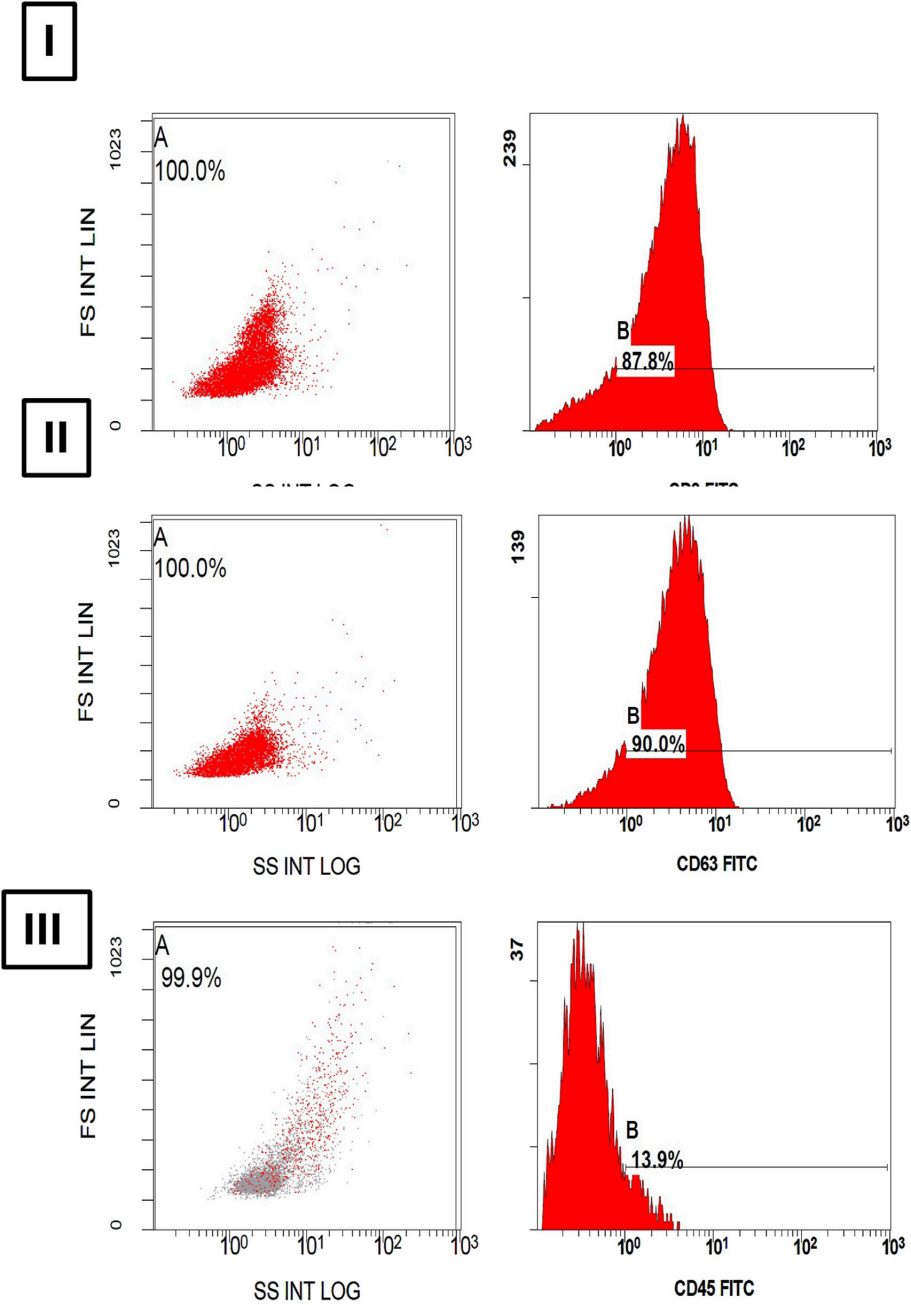
Histograms of **(A)** Dot-plot and **(B)** fluorescence intensity show a bright expression of **(I)** CD9 and **(II)** CD63 in 87.8% and 90.0% of extracellular particles as exosome specific membrane markers. However, a dim expression of **(III)** CD45 in 13.9% of extracellular particles is noted.

As displayed in Figure 2, the dot plot graphs illustrate the expression levels of each marker. Flowcytometry analysis showed positive expressions of (I) CD9, and (II) CD63 markers showing 87.8% and 90.0%, respectively, while the expression of (III) CD45 was unmarked displaying only 13.9%. Together, these findings reflect successful pure exosome isolation.

### 3.3 Treatment with MSC-derived exosomes regimens improved RTBI-induced behavioral alterations in NOR and Y maze tests

**FIGURE 3:**
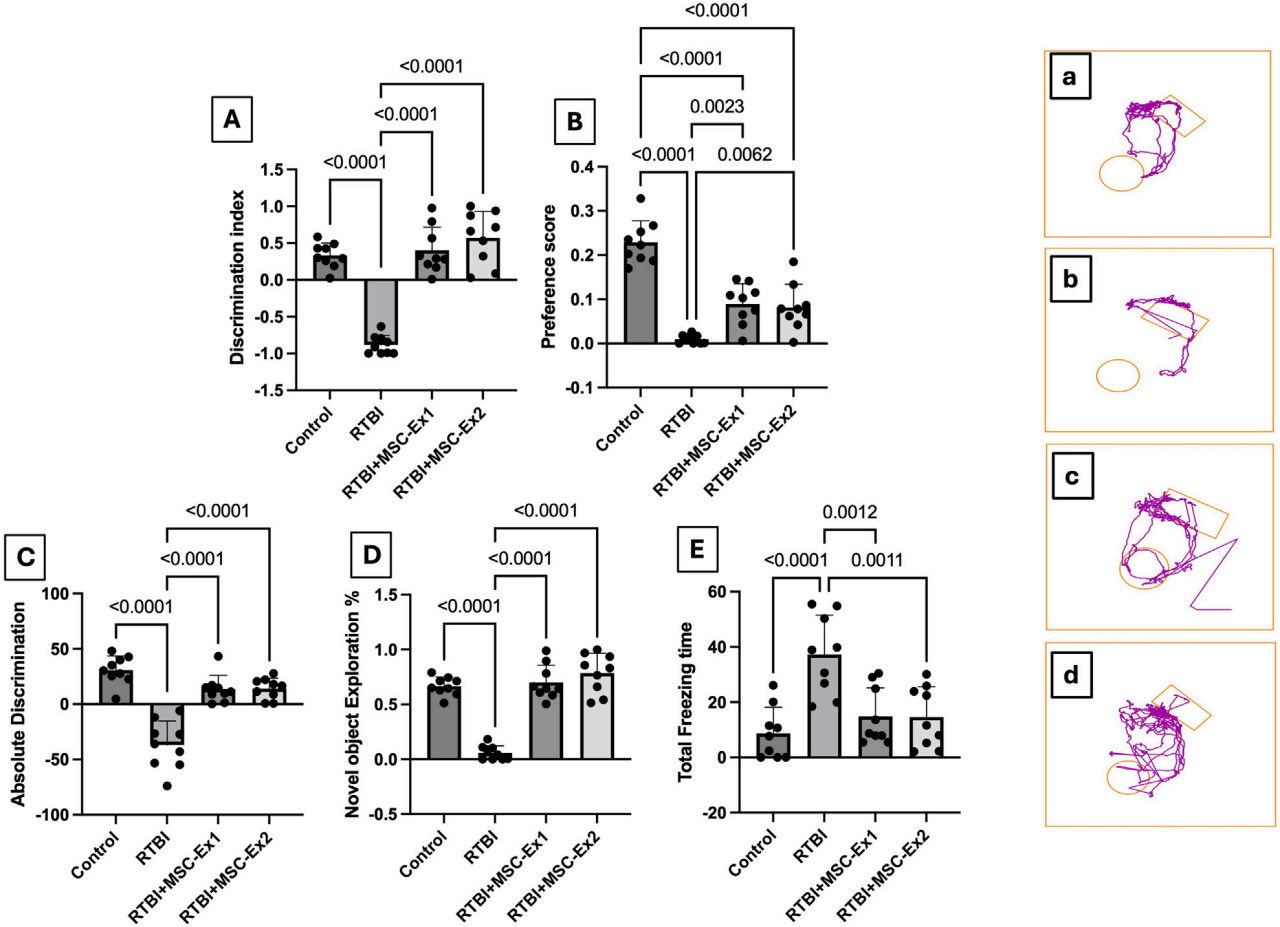
Assessment of **(A)** discrimination index, **(B)** preference score, **(C)** absolute discrimination, **(D)** novel object exploration %, and **(E)** total freezing time, as well as **(a–d)** representative Any-Maze captured movement traces of rats from different experimental groups. Scatter plot of nine animals displaying mean values ±SD. Statistical evaluation was conducted using one-way ANOVA, with Tukey’s *post hoc* analysis, considering a significance threshold of P < 0.05. The right panels **(a–d)** display the movement of animals toward the rectangular object (familiar) and/or circular one (novel). MSC: mesenchymal stem cell; MSC-Ex1: single administration of MSC-derived exosomes, given 24 h after the final trauma; MSC-Ex2: dual administration of MSC-derived exosomes, first dose given at 24 h post-final trauma, and a second dose on day 7 post-trauma; RTBI: repetitive traumatic brain injury.

Applying RTBI is associated with marked behavioral perturbations in the experimental cohort, as delineated in Figure 3. In this context, the RTBI group exhibited a negative (A) discrimination index, indicating a heightened preference for the familiar object compared to the normal control group, which displayed a significantly elevated discrimination index, proportionate to an extended interaction with the novel object relative to total exploration time. To further substantiate these findings, a similar trend was observed in the (B) preference score, which quantifies the proportion of time dedicated to novel object exploration relative to the total exploration duration. Additionally, RTBI-subjected rats exhibited a profound decline in (C) absolute discrimination and (D) novel object exploration %, in contrast to the control cohort, which demonstrated positive discrimination and a robust capacity to differentiate between familiar and novel objects. Consequently, as a downstream behavioral consequence, a spontaneous yet predictable prolongation in the freezing/immobility state, persisting for approximately 50 s, is depicted in panel (E) for the RTBI group compared to the uninjured control. Notably, to emphasize their neurorestorative prowess, both single and dual administrations of Ex comparably restored the discrimination index, and preference scores, augmenting absolute discrimination and novel object exploration %, while significantly reducing the immobility period, restoring it to baseline levels. All these behavioral modifications are further corroborated by the extracted panels (a-d) using the Any-Maze software analyzer.

**FIGURE 4:**
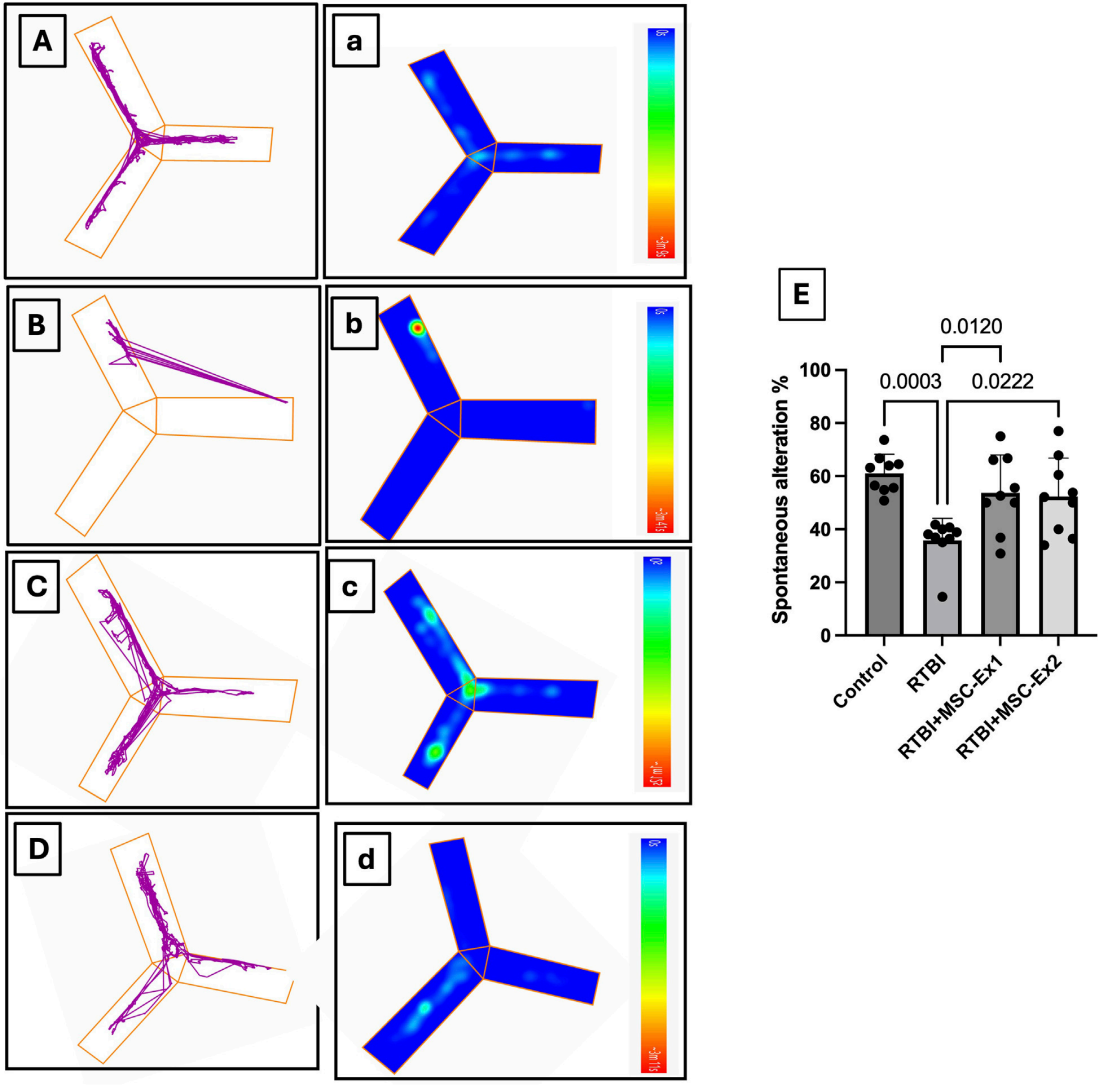
Assessment of spontaneous alternation behavior in the Y-maze following RTBI induction in a rat model. **(A–D)** The locomotor patterns within the maze and their **(A–D)** corresponding thermal maps illustrate that **(A,a)** control animals exhibit normative spontaneous alternation behavior with balanced arm traversal, whereas **(B,b)** the RTBI group demonstrates attenuated exploratory propensity, reduced alternations, and heightened immobility. Conversely, both the **(C,c)** RTBI + MSC-Ex1 cohort and **(D,d)** RTBI + MSC-Ex2 group exhibit a resurgence in alternation frequency alongside partial restoration of exploratory behavior that non-significantly emulates the control cohort, showcasing superior enhancement in alternation compared to the untreated RTBI group. Panel **(E)** presents a quantitative depiction of spontaneous alternation percentages (mean of 9 rats/group ±SD) across experimental groups, statistically analyzed via one-way ANOVA with Tukey’s *post hoc* test, establishing significance at P < 0.05. MSC: mesenchymal stem cell; MSC-Ex1: single administration of MSC-derived exosomes, given 24 h after the final trauma; MSC-Ex2: dual administration of MSC-derived exosomes, first dose given at 24 h post-final trauma, and a second dose on day 7 post-trauma; RTBI: repetitive traumatic brain injury.

Additionally, using the Y-maze test, which is a key paradigm in TBI for evaluating cortical integrity, essential for spatial navigation and decision-making, Figure 4 traces spatial memory by analyzing the innate exploration willingness of rats to a novel environment and the impact of RTBI on it. Panels (A-D) illustrate movement trajectories, while (a-d) depict heat maps that visualize spatial distribution pattern by mapping the activity of animals across different zones. Compared to the (A and a) healthy uninjured control cohort, (B and b) traumatized rats showed marked slow locomotion activity, with movement confined predominantly to a single arm, indicative of diminished exploratory drive and cortical dysfunction. Conversely, animals administered a single-dose MSC-Ex (C and c) 24 h post the last trauma displayed heightened activity, as evidenced by intensified green-yellow regions, signifying greater engagement with specific zones relative to the untreated RTBI cohort. While the double-dosed MSC-Ex rats **(D and d)** with a 1-week interval showed a uniform activity akin to the **(A and a)** control group highlighting the potential of repeated exosome administration in restoring cortical functionality. Panel **(E)** presents the quantitative analysis of Y-maze data, expressed as spontaneous alternation percentage (SA%). Repetitive trauma significantly impaired short-term spatial memory, as indicated by a reduced SA% compared to the normal levels observed in the healthy control group. Conversely, both MSC-derived exosomes treatment regimens preserved spatial memory, demonstrating a notable increase in SA% relative to the untreated RTBI group.

### 3.4 Histopathological alterations

**FIGURE 5:**
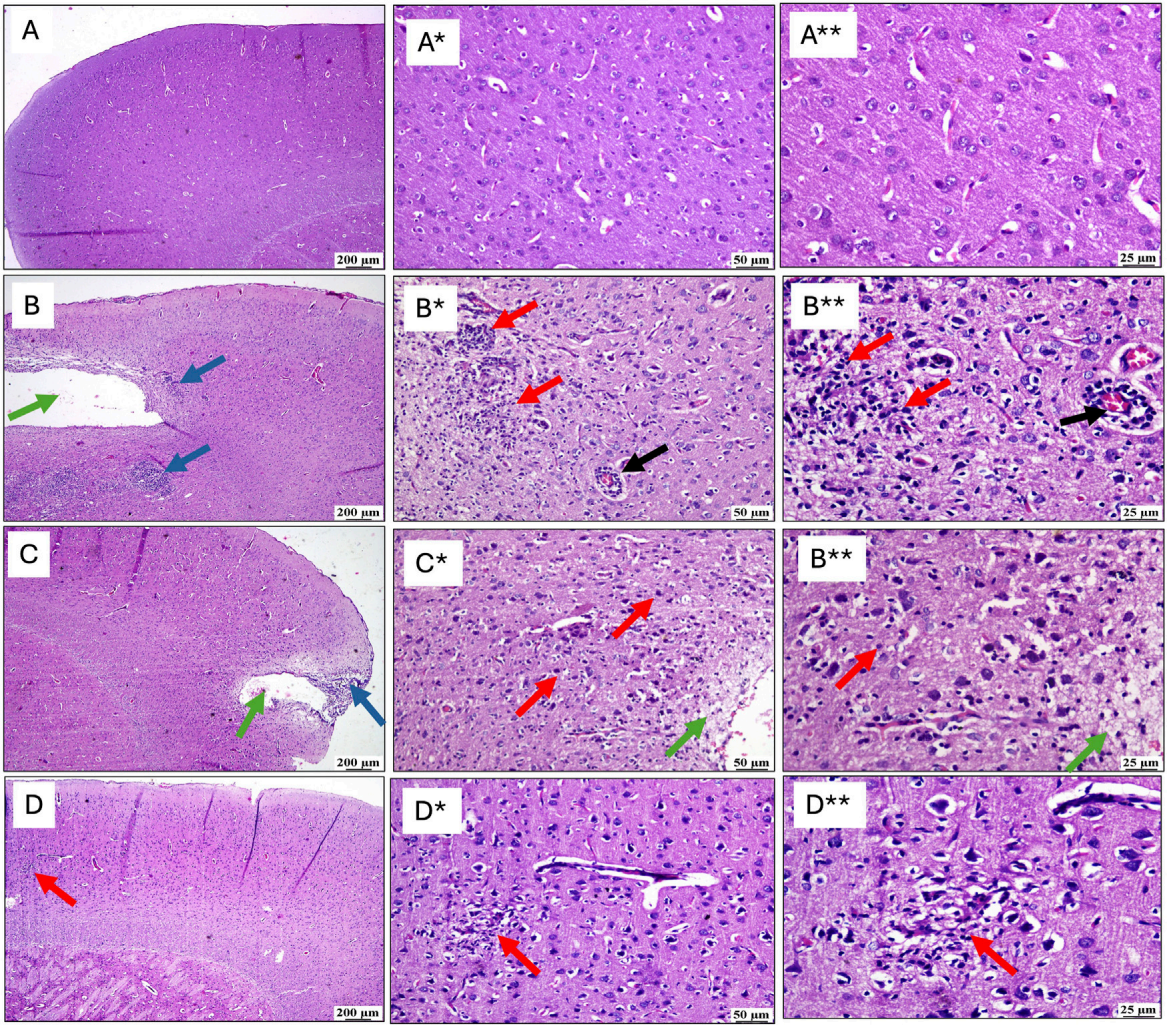
Effect of post-treatment with single or double doses of MSC-derived exosomes on cortical microscopical alterations following RTBI induction in a rat model (H&E stain; scale bar 200, (*) 50, and (**) 25 μm, respectively). Photomicrographs of the cerebral cortex from **(B)** the RTBI group demonstrate significant cortical loss (green arrow) along with a pronounced inflammatory response in the adjacent tissue (blue arrows), compared to the normal cerebral cortex of the **(A)** control cohort. Higher magnification images of (A*, A**) in the control group show intact tissue, while those (B*, B**) from the RTBI group reveal areas of malacia, gliosis (red arrows), and perivascular lymphocytic infiltration (black arrows). **(C)** The RTBI + MSC-Ex1 group exhibits small focal cortical loss (green arrow) and surrounding inflammatory changes (blue arrows), with higher magnifications (C*, C**) showing localized loss (green arrows) with gliosis (red arrows). Finally, the **(D)** RTBI + MSC-Ex2 group displays an apparently normal cerebral cortex, with only minor, scattered areas of malacia and gliosis (red arrows) as seen in higher magnification (D*, D**). MSC: mesenchymal stem cell; MSC-Ex1: single administration of MSC-derived exosomes, given 24 h after the final trauma; MSC-Ex2: dual administration of MSC-derived exosomes, first dose given at 24 h post-final trauma, and a second dose on day 7 post-trauma; RTBI: repetitive traumatic brain injury.

Figure 5 compiles the photomicrographs captured during the histopathological examination, with a comparative analysis against the normal cerebral cortex architecture of the control group (A, A*, A**). The cerebral cortex sections from the untreated RTBI group (B, B*, B**) revealed marked tissue loss, accompanied by intense inflammatory reactions in the adjacent areas, extensive malacia, severe diffuse gliosis, and perivascular lymphocytic infiltrates. In contrast, animals treated with (C, C*, C**) a single intravenous dose of exosomes demonstrated moderate recovery with the examined sections revealing localized cortical tissue loss and mild gliosis in the surrounding areas. Notably, the group receiving (D, D*, D**) two doses of exosomes showed substantial improvement, as the cortical sections displayed only small, sporadic focal areas of malacia with gliosis, surrounded by seemingly normal cerebral cortex tissue.

### 3.5 MSC-derived exosomes halt excitotoxicity and ROS production after RTBI induction in rat model

**FIGURE 6:**
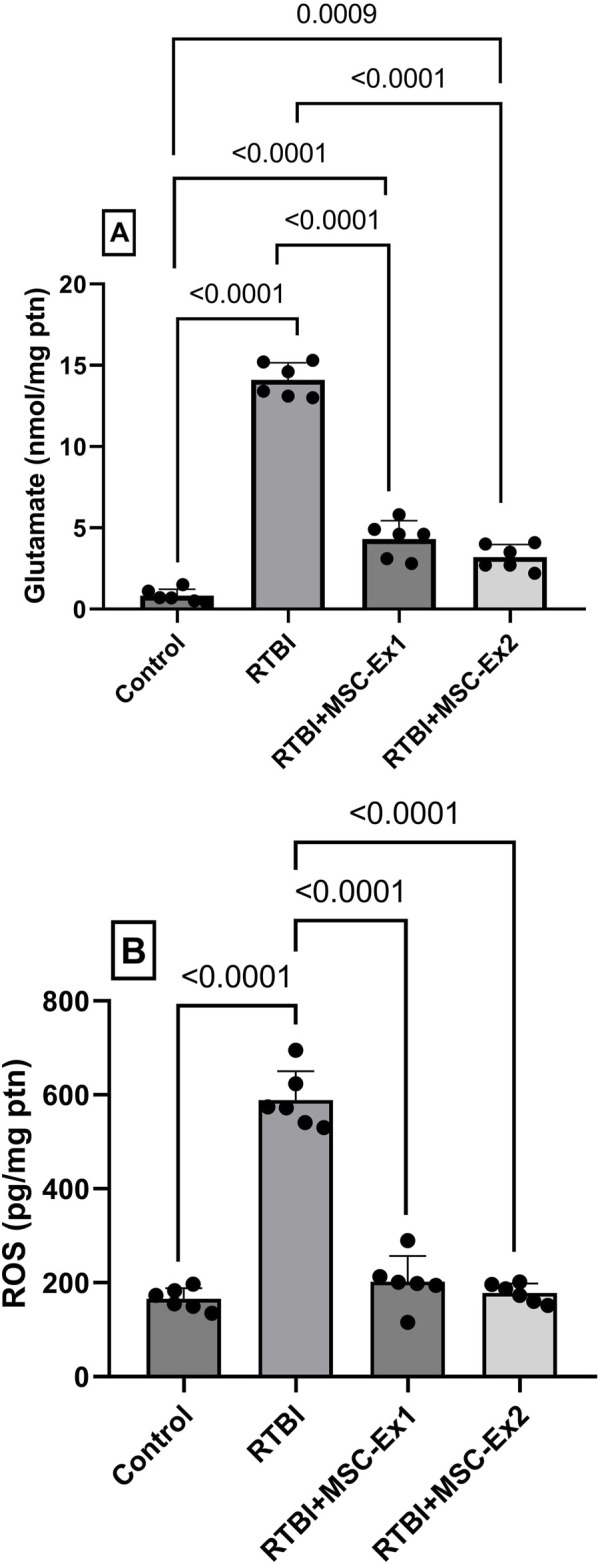
Effect of single or double doses of MSC-derived exosomes on cortical **(A)** glutamate and **(B)** ROS contents in rats subjected to RTBI. Scatter plot of six animals displaying mean values ± SD. Statistical evaluation was conducted using one-way ANOVA, with Tukey’s *post hoc* analysis, considering a significance threshold of P < 0.05. MSC: mesenchymal stem cell; MSC-Ex1: single administration of MSC-derived exosomes, given 24 h after the final trauma; MSC-Ex2: dual administration of MSC-derived exosomes, first dose given at 24 h post-final trauma, and a second dose on day 7 post-trauma; ROS: reactive oxygen species; ptn: protein; RTBI: repetitive traumatic brain injury.

The RTBI aptitude to augment injurious mediators is delineated in Figure 6. RTBI insult is known to trigger excitotoxicity as evidenced in panel (A) by the substantial bolstering in cortical glutamate content (17-fold) in traumatized rats. Moreover, this detriment also prompts the formation of ROS as displayed in panel (B), which reveals a 3.7-fold elevation in ROS production, underscoring a perceptible OS response. In contrast, both MSC-derived exosomes treatments effectively mitigated these pathological alterations, quelling glutamate content markedly while restoring ROS levels to near-normal values. Although the two-dose regimen showed a subtle improvement compared to the single one, this effect did not reach statistical significance, further highlighting the similar efficacy of both treatments in preventing cortical damage induced by RTBI.

### 3.6 MSC-driven exosomes alleviate cortical PARP1 overactivation, PAR formation, and NAD + consumption in RTBI-induced parthanatos

**FIGURE 7:**
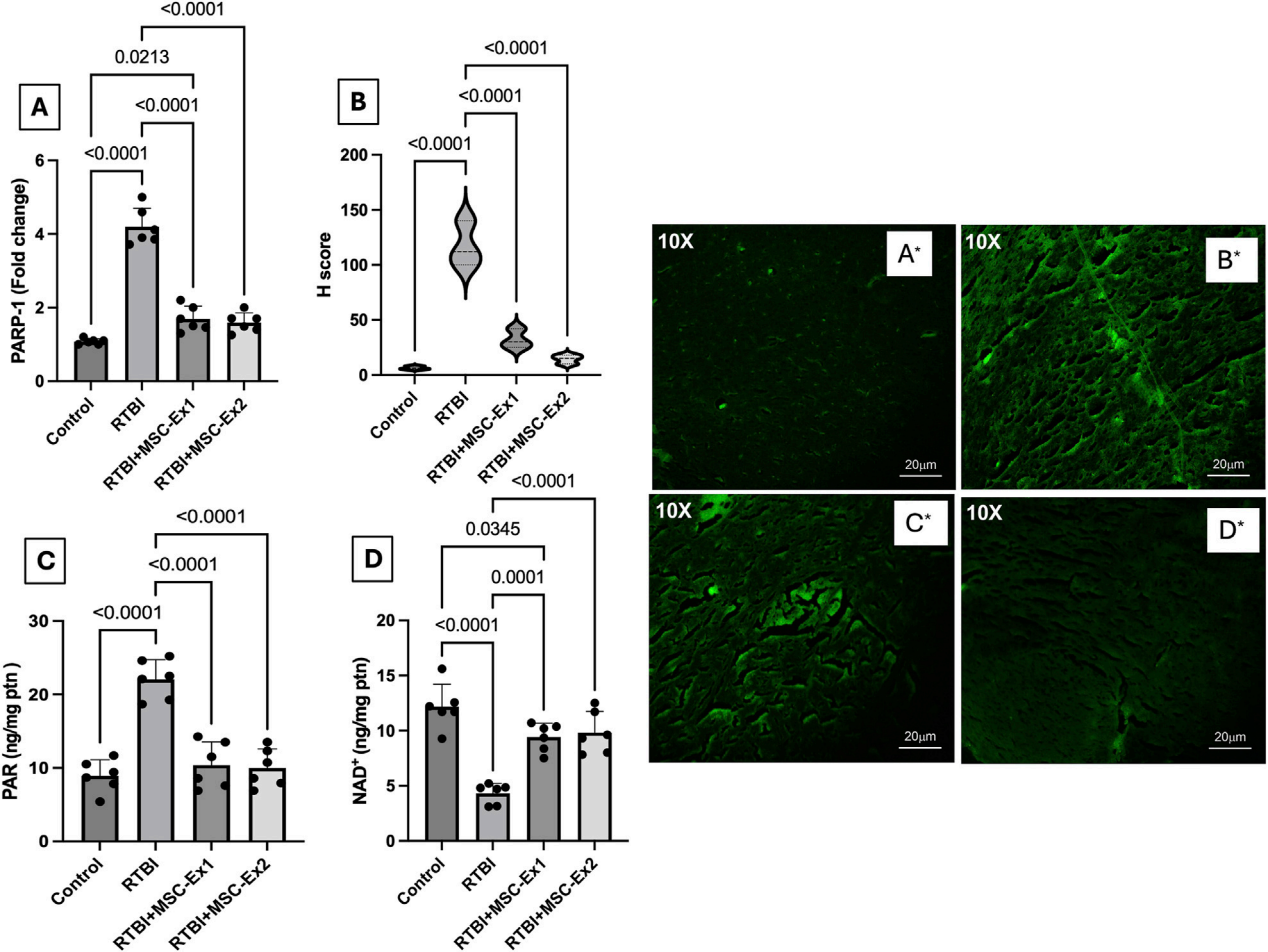
Impact of single or double doses of MSC-derived exosomes on cortical **(A)** PARP1 mRNA expression, **(B)** H-score analysis of PARP1 protein expression based on (A*–D*) PARP1 immunofluorescence-stained photomicrographs [10X, 20 µm scale bar], and cortical contents of **(C)** PAR, and **(D)** NAD+ in rats with RTBI. The (B*) RTBI section shows a strong expression of PARP1 (fluorescence intensity +3) as compared to the control tissue that reveals a weak expression of PARP1 with mild fluorescence intensity (+). On the treatment side, the tissue section of RTBI + MSC-Ex1 group presents a mild to moderate expression of PARP1 with moderate fluorescence intensity (+2), whereas the RTBI + MSC-Ex2-treated group depicts a mild expression of PARP1 (+1). Parametric data are presented as a scatter plot of six animals displaying mean values ±SD. Statistical evaluation was conducted using one-way ANOVA, with Tukey’s *post hoc* analysis, considering a significance threshold of P < 0.05. MSC: mesenchymal stem cell; MSC-Ex1: single administration of MSC-derived exosomes, given 24 h after the final trauma; MSC-Ex2: dual administration of MSC-derived exosomes, first dose given at 24 h post-final trauma, and a second dose on day 7 post-trauma; NAD: nicotinamide adenine dinucleotide; PARP1: poly-ADP ribose (PAR) polymerase-1; ptn: protein; RTBI: repetitive traumatic brain injury.

The impact of RTBI on the parthanatos cascade is illustrated in Figure 7. As a consequence of excessive ROS generation, DNA damage ensued, culminating in the hyperactivation of the parthanatos hallmark enzyme, (A) PARP1, whose RNA expression surged by 310% following RTBI. This transcriptional upregulation was further corroborated by immunofluorescence staining (A*–D*), which revealed marked PARP1 protein overexpression in cortical tissue (B*) following RTBI. Quantitative analysis using the (B) H-score method demonstrated an 18.5-fold increase in cortical PARP1 expression in the RTBI group. This overactivation led to a 147% elevation in cortical levels of its downstream effector (C) PAR, alongside a significant depletion of its essential cofactor (D) NAD^+^, which dropped to just 35.5% of control values. However, treatment with either (C*) single or (D*) dual doses of MSC-derived exosomes significantly attenuated RTBI-induced parthanatos, as evidenced by reduced PARP1 expression. Although the dual-dose regimen achieved a greater reduction in PARP1 levels compared to the single dose (14.3 ± 4 vs. 32.3 ± 8.7) and was statistically indistinguishable from the control group, the difference between the dual- and single-dose groups did not reach statistical significance. The PARP1 downregulation was associated by the subsequent restraint of PAR formation, effectively preserving NAD + levels. Notably, these restorative effects closely approximated those observed in the uninjured control cohort.

### 3.7 MSC-driven exosomes blunt RTBI-mediated mitochondrial dysfunction and parthanatos: modulation of calpain, AIF, MIF, CYP B, and Hsp70

**FIGURE 8:**
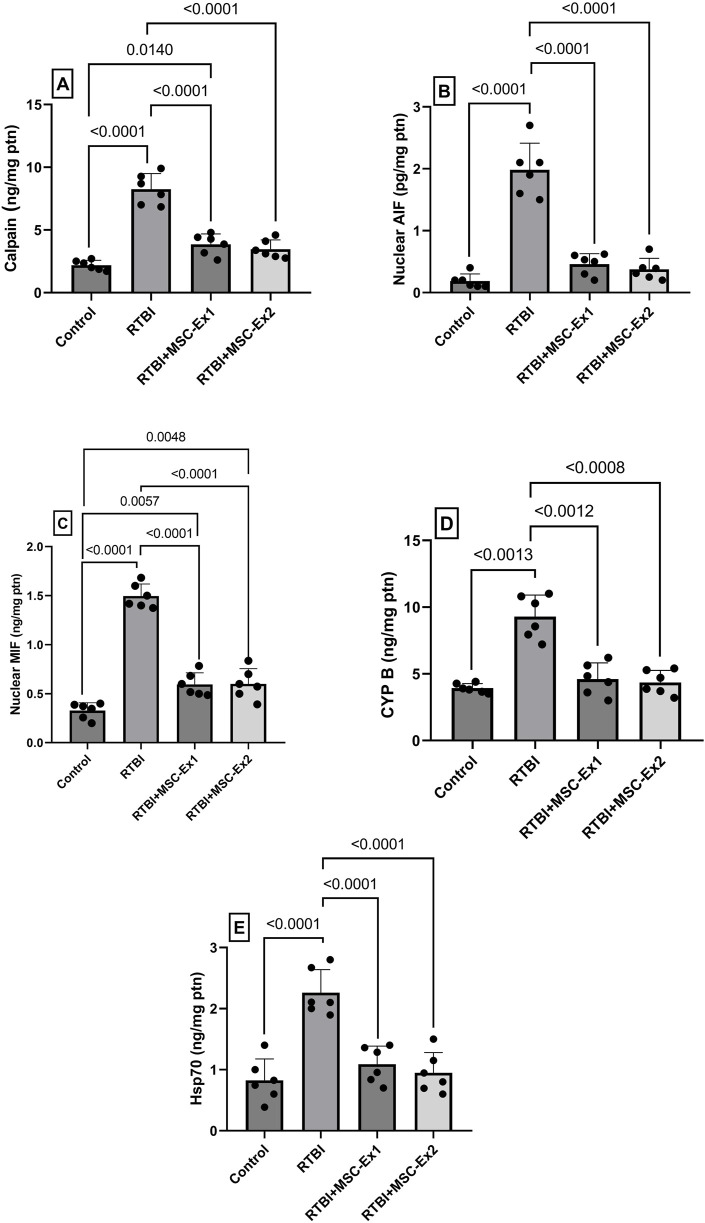
Impact of single and double doses of MSC-derived exosomes on cortical contents of **(A)** calpain, **(B)** AIF, **(C)** MIF, **(D)** CYP B, and **(E)** Hsp70 in rats with RTBI. Scatter plot of six animals displaying mean values ±SD. Statistical evaluation was conducted using one-way ANOVA, with Tukey’s *post hoc* analysis, except for CYP B which was analyzed using Welch’s ANOVA followed by Dunnett’s T3 Multiple Comparison as the *post hoc* test. Both tests consider the significance threshold of P < 0.05. AIF: apoptosis inducing factor; CYP (B) cycophilin B; Hsp70: heat shock protein 70; MIF: macrophage migration inhibitory factor; MSC: mesenchymal stem cell; MSC-Ex1: single administration of MSC-derived exosomes, given 24 h after the final trauma; MSC-Ex2: dual administration of MSC-derived exosomes, first dose given at 24 h post-final trauma, and a second dose on day 7 post-trauma; ptn: protein; RTBI: repetitive traumatic brain injury.

Following RTBI-induced excitotoxicity and OS, the ensuing cellular insult precipitated mitochondrial dysfunction and exacerbated parthanatos-mediated cell demise (Figure 8). Following enhanced excitotoxicity, the RTBI affront triggered a 4-fold surge in the cortical content of the calcium-activated protease (A) calpain accompanied by an (B) excessive AIF release and nuclear translocation, elevating its nuclear cortical content to 19-fold compared to the control group. Additionally, this was associated by a 5-fold upsurge of its complexing partner (C) MIF, culminating in further parthanatos progression. Intriguingly, the insult increased (D) CYP B, and (E) Hsp70 by a respective 2-fold and 2.6-fold possibly to counteract this deleterious cascade. However, administration of MSC-derived exosomes, either 24 h and/or 7 days post-trauma, almost halved calpain cortical concentration to correlate with a marked ablation of nuclear AIF and MIF content, thereby mitigating parthanatos-driven neurodegeneration. Additionally, the treatments restored the cortical contents of both CYP B and Hsp70, reflecting the diminished necessity for their protective function under exosome therapy.

### 3.8 MSC-driven exosomes subdue RTBI-induced inflammatory mediators

**FIGURE 9:**
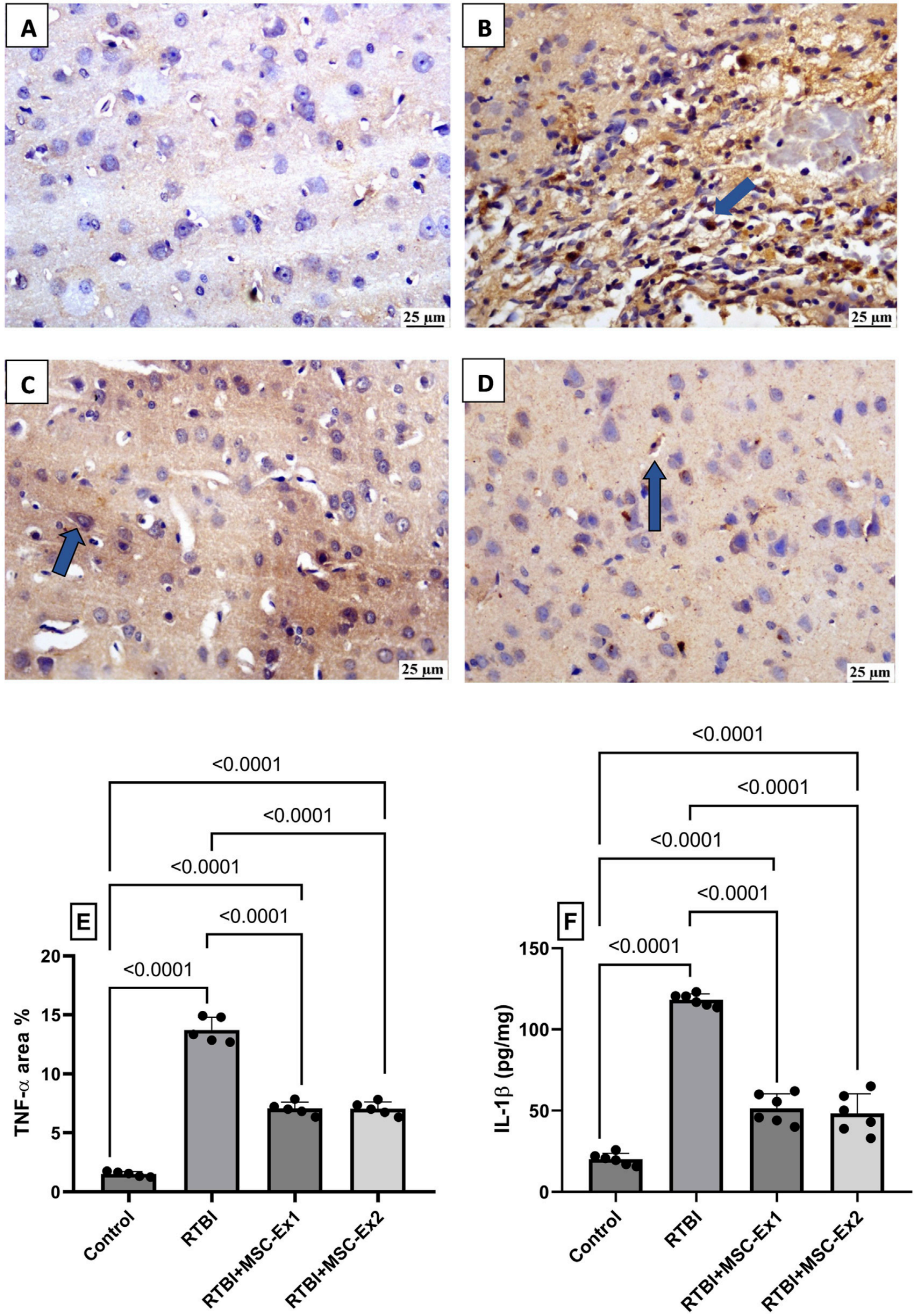
Impact of single and double doses of MSC-derived exosomes on cortical **(A–E)** immune-expression of TNF-α and (F) IL-1β content in rats with RTBI. The photomicrographs of cortical IHC of TNF-α in **(B)** RTBI group reveal intense positive immunostaining of the inflammatory mediator (blue arrow), compared to the naively presented **(A)** control group. Nevertheless, treatment with **(C)** MSC-Ex1 and **(D)** MSC-Ex2 show significant reduction in protein expression of TNF-α with approximately equal positive nuclei expression (blue arrow). As visualized in panel **(E)**, scatter plot of 5 fields from 3 rats/group displays mean values of area percentage of TNF-α ± SD. In panel **(F)**, scatter plot from six animals represents cortical contents of IL-1β, in which values are shown as mean of 6 samples ±SD. For both parameters, statistical evaluation was conducted using one-way ANOVA, with Tukey’s *post hoc* analysis, considering a significance threshold of P < 0.05. IL-1β: interleukin-1 beta; MSC: Mesenchymal stem cell; MSC-Ex1: Single administration of MSC-derived exosomes, given 24 h after the final trauma; MSC-Ex2: Dual administration of MSC-derived exosomes, first dose given at 24 h post-final trauma, and a second dose on day 7 post-trauma; RTBI: repetitive traumatic brain injury; TNF-α: tumor necrosis factor-alpha.

As illustrated in Figure 9, RTBI incites robust inflammatory cascades, as evidenced in panel (B), which exhibits pronounced protein expression of the pro-inflammatory cytokine TNF-α, surging to a (E) 9-fold increase compared to the (A) naïve group, where TNF-α expression is virtually undetectable. Highlighting their anti-inflammatory potential, sections (C and D) display attenuated immunohistochemical staining of TNF-α, reducing its expression by nearly half relative to the RTBI group. As depicted in panel (E), both MSC-derived exosomes regimens effectively mitigated TNF-α expression, normalizing it to 51% of RTBI levels. Similarly, RTBI provoked a substantial elevation in **(F)** IL-1β, amplifying its cortical content nearly 6-fold compared to the control cohort. However, post-treatment with MSC-derived exosomes regimens significantly curtailed IL-1β cortical contents, yielding a 56% and 60% reduction in single-dose MSC-Ex and double-dose MSC-Ex groups, respectively, relative to the untreated RTBI group.

## 4 Discussion

In line with their neuroprotective potential, the ongoing inquiry highlights for the first time the neurotherapeutic proficiency of MSC-derived exosomes in alleviating RTBI-induced parthanatos-mediated programmed neuronal demise in rats. Through comprehensive analyses, we substantiate that both MSC-derived exosomes regimens comparably fortified the brain against RTBI-evoked neurodegenerative sequelae, as evidenced by the restitution of spatial memory and the conservation of cortical histoarchitecture. In part, these enhancements can be attributed to the inhibition of the triadic neurotoxic nexus; *viz.,* excitotoxicity, OS, and parthanatos cell death. At the molecular level, the treatment regimens dampened cortical glutamate and ROS levels, thereby hindering PARP1 overexpression and the subsequent surge in its downstream effector, PAR, ultimately resulting in NAD + replenishment. Additionally, disrupting this neurotoxic triad declined the protease enzyme calpain, which in turn with the inhibited PAR impeded the release and nuclear translocation of AIF, as well as MIF, thereby obstructing the formation of their deleterious complex that threatens DNA repair. Interestingly, our study confirmed that exosomes treatment restored the two molecular chaperones CYP B, which is studied for the first time in this ailment along with Hsp70, which were bolstered in the untreated RTBI animals, possibly to play a compensatory role against the current detriment. Finally, by thwarting RTBI-induced neurodegenerative cascades, MSC-derived exosomes post-treatment attenuated neuroinflammation, as evidenced by a reduction in cortical immunoreactivity of TNF-α and a diminished IL-1β concentration, further affirming the anti-inflammatory potency of MSC-derived exosomes.

In evaluating the physicochemical characteristics of exosomes, it is important to consider methodological differences in size measurements. Discrepancy between TEM and DLS is not uncommon; TEM provides a snapshot of vesicle morphology under dehydrated conditions, often yielding smaller sizes, while DLS measures the hydrodynamic diameter in solution, which may appear larger due to the presence of surface-bound proteins or other biomolecules. A consistent size difference of approximately 40 nm between DLS and TEM has been reported (Lyu et al., 2021), and similar findings have been documented in other studies (Chernyshev et al., 2015; Sokolova et al., 2011), reinforcing that such differences stem from the techniques used rather than variations in exosome quality.

The observed phenotype, CD45^-^/CD9^+^/CD63^+^, indicates a pure population of isolated exosomes. CD9 and CD63, members of the tetraspanin family found on the exosomal surface, are not directly therapeutic themselves but play a pivotal role in mediating the therapeutic potential of exosomes. Their expression is associated with key functions such as cellular targeting, exosome stability, and immune modulation. These features make them vital for exosome-based interventions in neurodegenerative disorders, including TBI. Accordingly, exosomes enriched with the identified CD markers can serve as carriers for neuroprotective cargos, including specific microRNAs, various proteins, and anti-inflammatory cytokines, capable of crossing the BBB to target affected neurons (Dehghani et al., 2025; Jankovičová et al., 2020). This targeted delivery may impede disease progression by attenuating inflammation and enhancing neuronal survival in neurodegenerative conditions (Nouri et al., 2024; Zhang et al., 2024).

In the current work, the NORT substantiated the therapeutic capacity of MSC-derived exosomes in alleviating RTBI-induced aberrant behavioral response, which diminished exploratory proclivities and engagement with novel objects corroborating previous observations in various neurological disorders (Fallahi et al., 2024; Jovanovic et al., 2018). The memory restitution prowess of MSC-derived exosomes therapy was further validated by the Y-maze results reported herein. The behavioral anomalies alongside the changes in cortical architecture depicted in the RTBI-injured animals, may be ascribed not only to the primary mechanical trauma from the weight drop, resulting in disrupted brain architecture, but also to the second sequelae of trauma. Hence, from a biomechanical perspective, RTBI was associated with a pronounced surge in the neurotoxin glutamate and aggravated oxidative duress, phenomena validated in the present study and corroborated by prior studies (Mckee and Daneshvar, 2015) playing a cardinal role in precipitating subsequent deleterious cascades.

Accordingly, the neurotherapeutic capacity of MSC-derived exosomes regimens to ameliorate behavioral and histopathological deficits can be partially accredited to their ability to diminish cortical glutamate content, reinforcing an earlier observation involving MSC-driven exosomes in mild single-impact brain trauma (Zhuang et al., 2022). Despite the renowned nexus between OS and RTBI pathophysiology (El-Gazar et al., 2024; Zink et al., 2010), no prior evidence has chronicled the antioxidant prowess of MSC-driven exosomes in repetitive TBI models. Nevertheless, their competency in mitigating OS has been reported in alternative central nervous system injury paradigms (Quan et al., 2025; Xie et al., 2023; Zaazaa et al., 2021), where they contributed to cognitive enhancement, spatial memory consolidation, and augmented exploratory behavior. This reparative aptitude of exosomes is largely attributed to their potent intercellular signaling capabilities and their cargo of bioactive mediators (Y. Chen et al., 2020; Reza-Zaldivar et al., 2019).

The TBI-induced free radicals (FRs) generation and ROS overproduction is mediated primarily via mitochondrial disruption (Besson, 2009). This oxidative assault damages cellular components, including DNA, lipids, and proteins. Substantial DNA cleavage has been reported post-TBI (Davis and Vemuganti, 2021; LaPlaca et al., 1999; Schwab, Tator and Hazrati, 2019), leading to the activation of PARP1 as a reparative attempt. However, under excessive genotoxic stress, PARP1 becomes pathologically hyperactivated, exacerbating cellular damage (Besson, 2009; Huang et al., 2022). This dysregulation has been associated with cognitive impairments observed both in this study and in previous TBI models, where PARP1 inactivation alleviated such deficits (Whalen et al., 1999).

The neuroprotective efficacy of MSC-derived exosomes primarily hinges on the marked downregulation of PARP1 overexpression, an adjustment that was paralleled by a considerable increment in NAD^+^ levels to aid in mitigating neurodegeneration. In fact, NAD^+^ serves as a crucial redox cofactor indispensable for preserving mitochondrial bioenergetics by fine-tuning the generation and scavenging of ROS. Moreover, NAD^+^ orchestrates an array of essential cellular processes, including DNA repair, metabolic equilibrium, cell cycle regulation, protein-protein signaling, and modulation of inflammatory cascades (Martínez-Morcillo et al., 2021). As reflected in our findings, RTBI-provoked upregulation of PARP1 culminated in NAD^+^ attrition, a cardinal event in the parthanatos paradigm, wherein excessive NAD^+^ consumption during abortive DNA repair triggers energetic collapse and exacerbates mitochondrial impairment that propels cell demise (Murata et al., 2019). In tandem, NAD^+^ paucity contributes to unchecked ROS accumulation, perpetuating a vicious cycle of oxidative damage (Ying, 2013). In support, previous studies have documented PARP-driven NAD^+^ exhaustion in hippocampal and cortical regions post-trauma (Y. Lai et al., 2008) to brace our current data.

The function of the PARP1 enzyme also governs PAR catabolism and must be meticulously regulated to prevent either excessive PAR buildup or untimely PAR breakdown. However, in case of hyperactivated PARP1 and energy depletion, the metabolically exhaustive cycle is catalyzed, generating an overabundant PAR polymer that translocates to cytoplasm and mitochondria, where it interacts with AIF, triggering its release (Y. Wang et al., 2011). In the cytoplasm, AIF engages in a molecular liaison with the nuclease protein MIF, enlisting it as a nuclear chaperone to facilitate its translocation into the nucleus, where MIF slices genomic DNA into substantial segments and exacerbates cellular damage (Huang et al., 2022)As a result of the thwarted glutamate/ROS/PARP1, and the replenishment of NAD+, MSC-derived exosomes therapy, restored cortical content of PAR, as well as the nuclear level of both AIF and MIF, pointing for the possible DNA repair. In concordance with the significant ablation of the AIF and MIF deleterious molecules, Wang et al. (2016) demonstrated that the genetic abrogation of MIF or perturbation of the AIF-MIF intricate nexus nullified its endonuclease aptitude, thereby impeding glutamate-induced excitotoxic neuronal demise in a focal cerebral ischemia model.

In our study, MSC-derived exosomes effectively suppressed elevated cortical calpain levels, aiding in the restoration of cellular homeostasis. Beyond glutamate-induced Ca^2+^-driven FRs generation, recent evidence suggests that Ca^2+^ signaling also activates PARP1 (Zhou et al., 2021), partly via Ca^2+^-dependent proteases like calpains (Besson, 2009), a finding consistent with our RTBI model. In fact, activated calpain contributes significantly to neuronal injury by degrading key neuronal proteins (Metwally et al., 2023), disrupting the nuclear envelope (Singh et al., 2024), and cleaving mitochondrial-protective proteins, thus promoting mitochondrial dysfunction (Shan et al., 2022). It also facilitates AIF maturation and its mitochondrial egress (Cao et al., 2007). However, whether calpain inhibition genuinely enhances cell survival in PARP1-driven cell death or merely delays AIF translocation remains uncertain (J. Zhang et al., 2025). Regardless, MSC-derived exosomes mediated inhibition of calpain likely contributed to attenuating parthanatos-associated neurodegeneration.

Our study assessed for the first time the potential impact of RTBI and MSC-derived exosomes on the molecular chaperone CYP B. Our findings showed that treatment with MSC-derived exosomes succeeded in restoring CYP B to its normal level after being boosted in the RTBI model. Indeed, CYPs constitute a subset of immunophilin proteins that exhibit peptidyl-prolyl cis-trans isomerase (PPIase) catalytic functionality and are implicated in diverse cellular processes, including protein folding, signal transduction, intracellular trafficking, mitochondrial preservation, and transcriptional regulation (Andreeva et al., 1999; Gegunde et al., 2021; Liang et al., 2021). The CYP B chaperon is distinctive due to possessing an endoplasmic reticulum (ER)-targeting signal sequence, though it is also present in the extracellular space and the nucleus (Oh et al., 2011). Despite the scarce data on the role of CYP B in neurodegenerative diseases, an earlier *in-vitro* study pointed to the ability of overexpressed CYP B to attenuate PARP cleavage, DNA disintegration, and ROS-induced neurotoxicity mediated by beta amyloid 25–35 model (Oh et al., 2011). Subsequently, in an Alzheimer’s disease paradigm, the investigators disclosed that suppression of the ER-resident chaperone, CYP B, instigates presenilin accrual concomitant with mitochondrial perturbation (Ben-Gedalya et al., 2015). Concordant with the neuroprotective capacity attributed to CYP B, Zhuang et al. (Zhuang et al., 2022) reported its involvement in the intracellular internalization of nucleic acids. Given the salutary attributes of CYP B, we posited that its RTBI-induced stimulation may serve a compensatory function counteracting the neurodegenerative sequelae of RTBI. Supporting this premise, Wang et al. (B. Wang et al., 2016) documented that CYP B overexpression ameliorated aldosterone-evoked nephrotoxicity by enhancing mitochondrial performance and curbing ROS accumulation. Intriguingly, and to support our findings, these authors reported bolstered levels of the molecular chaperone in the aldosterone-induced kidney injury model, implying a compensatory response in renal tubular epithelia. Since MSC-derived exosomes were reported to reduce protein misfolding and enhance mitochondria function indeed by its current aptitude to preserve NAD^+^ with its critical anti-parthanatos efficacy could be the culprits for the reduced CYP B cortical content observed in our study.

Another molecular chaperone that was boosted in RTBI cohort is Hsp70 that governs proteostasis by balancing protein proper folding, functional regulation, and catabolism to mediate its chaperone therapeutic impact (Kim et al., 2020). Being a stress-inducible effector, Hsp70 provokes its survival potential across diverse pathological milieus, including tissue injury, ischemia-reperfusion insult, and inflammatory states (Zhao et al., 2013). Additionally, Hsp70 cytoprotective actions are enacted through attenuating intrinsic apoptotic signaling, preserving mitochondrial integrity (Tsuchiya et al., 2003), stifling Ca^2+^ intracellular accumulation in a model of ischemia/reoxygenation (Liu et al., 2016) hampering thus cellular injury. These effects can partake in the Hsp70 anti-parthanatos capacity, besides its ability to interlock AIF, thereby restraining its nuclear translocation (Matsumori et al., 2005). Furthermore, Hsp70 tempers caspase-independent demise, where it was found to exhibit nuclear and nucleolar co-localization with PARP1, a molecular interplay that implies a synergistic involvement in orchestrating DNA repair pathways and mediating cellular adaptive responses under genotoxic or stress-inducing conditions (Kotoglou et al., 2009). To further signify its neuroprotective effect, exogenous augmentation of Hsp70 expression has been shown to diminish neuronal attrition in experimental paradigms of cerebral ischemia and in kainic acid-induced excitotoxicity. In contrast, Hsp70-deficient murine models exhibit exacerbated cerebral infarction post-ischemia (Lee et al., 2001). In such contexts, the overexpressed Hsp70-mediated orchestration of key neurodegenerative cascades, may rationalize its heightened levels in stress, injured animals as a recuperative mechanism, supplementing the primary molecular chaperone CYP B.

Interestingly, treatment with MSC-derived exosomes attenuated the elevated levels of Hsp70 and CYP B observed in RTBI-exposed animals. This reduction may reflect a diminished cellular burden, potentially lowering the need for compensatory upregulation of these stress-related chaperones. However, the precise functional roles of Hsp70 and CYP B in the context of RTBI remain to be fully elucidated, and further studies are warranted to confirm this interpretation. In support of their potential cytoprotective capacity, MSC-derived exosomes are known to deliver functional proteasomes, including the 20S catalytic core, to injured tissues (R. C. Lai et al., 2012). This proteolytic complex plays a key role in maintaining proteostasis by degrading misfolded or aggregated proteins, thereby alleviating cellular stress (Pepelnjak et al., 2024; Yeh Yeo et al., 2013). Moreover, MSC-derived exosomes possess well-documented antioxidant (Elnegris et al., 2024), anti-inflammatory (Chen et al., 2024), and antiapoptotic (Zhuang et al., 2023) properties, which may collectively contribute to reducing the reliance on chaperone-mediated stress responses following RTBI.

Additionally, MSC-derived exosomes underscored their anti-inflammatory effect by curbing the protein expression of both TNF-α and IL-1β to align with an earlier study using MSC-derived exosomes in early-stage post single hit (Ni et al., 2019). In a recent study, Tang et al. (Tang et al., 2023) chronicled that exosomes derived from adipose-derived stem cells were able to quell both inflammatory markers in microglia exposed to lipopolysaccharide. RTBI-induced inflammation is the final detrimental pivot recorded herein to exacerbate neuronal injury and compromising neural repair mechanisms. This event has been documented by previous studies (Hiskens et al., 2021; J. Wang et al., 2025). Moreover, exosomes-mediated inhibition of the parthanatos axis further amplifies their robust anti-inflammatory efficacy. In this vein, PARP1 antagonists have been shown to markedly suppress inflammatory indices in TBI rat paradigms (d’Avila et al., 2012) and in a model of localized aseptic meningitis (Rom et al., 2016). Similarly, neuroinflammation was attenuated in a murine TBI model following administration of a parthanatos modulator (Shi et al., 2022) or calpain suppressor (Tao et al., 2017). A prior investigation also underscored the anti-inflammatory utility of NAD^+^ in shielding neural tissue subjected to TBI and ischemic insults (Ying, 2013).

## 5 Conclusion

This study unveils a promising therapeutic paradigm where MSC-derived exosomes confer robust neuroprotection against the multifaceted damage of RTBI. Through broad-spectrum modulation of parthanatos-induced cell death, and the secondary injurious mechanisms excitotoxicity, oxidative duress, and inflammation, MSC-derived exosomes significantly restored cognitive performance and preserved cortical structure. Mechanistically, they curbed glutamate and ROS levels, halting the parthanatos cascade by inhibiting PARP1, PAR, and the nuclear translocation of AIF and its nuclease partner MIF. Additionally, calpain suppression added a vital layer of mitochondrial and nuclear safeguarding. Intriguingly, MSC-derived exosomes rebalanced the heightened expression of CYP B and Hsp70, stress-responsive chaperones likely upregulated as part of an intrinsic compensatory defense response. These findings highlight the involvement of parthanatos in RTBI-associated neurodegeneration and provide a strong preclinical foundation for the translational application of MSC-derived exosomes as a novel therapeutic strategy in managing RTBI-associated neurodegeneration. Future research should refine delivery protocols and evaluate the potential of MSC-derived exosomes to influence long-term outcomes of RTBI, including chronic neurodegenerative changes, thereby helping to bridge the gap between bench and bedside.

## 6 Limitations

Despite the encouraging results, this study has several limitations. The relatively short follow-up period limits conclusions regarding the long-term efficacy and safety of MSC-derived exosomes in RTBI. Longitudinal studies are needed to assess the durability of neuroprotection and explore the potential of repeated dosing to prevent or delay chronic neurodegenerative outcomes. Additionally, the exclusive use of male Wistar rats restricts the generalizability of the findings across sexes and species. Furthermore, the behavioral assessment incorporating a broader battery of cognitive and motor evaluations would offer a more comprehensive view of the therapeutic impact. These considerations underscore the need for further research to validate and extend the current findings.

## 7 Future perspectives

Future studies should explore the long-term efficacy of MSC-derived exosomes in RTBI, particularly their potential to prevent chronic neurodegenerative outcomes such as tau and TDP-43 pathology. Investigating the role of parthanatos in chronic injury, optimizing dosing strategies, and evaluating alternative delivery routes will be essential for clinical translation. Standardization of human exosome preparations and comprehensive safety assessments, including immunogenicity and tissue distribution, will further enhance the therapeutic viability of this cell-free approach.

## Data Availability

The original contributions presented in the study are included in the article/Supplementary Material, further inquiries can be directed to the corresponding authors.
