# Supplementary figures and images for: Parthanatos drives cognitive decline in repeated brain trauma: MSC-derived exosomes as a novel therapeutic strategy
Source: Front Pharmacol. 2025 Sep 2;16:1622018. doi: 10.3389/fphar.2025.1622018 (PMC12436289; doi:10.3389/fphar.2025.1622018)

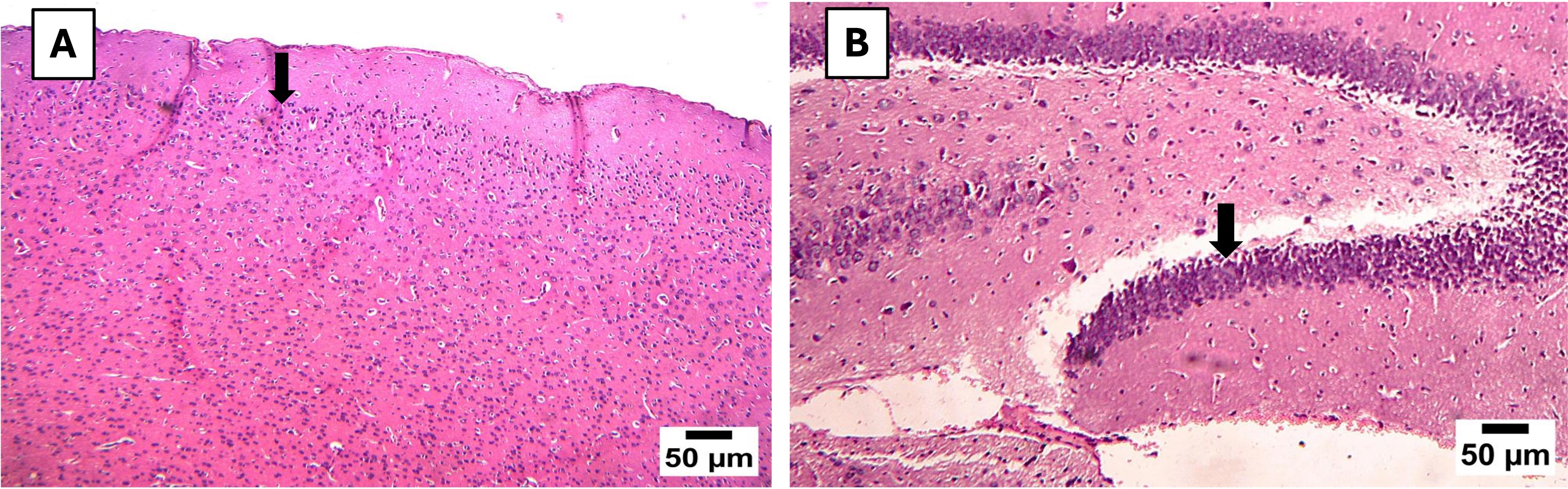

Supplement: Supplementary file 1 [file Image1.jpeg]
